# Preliminary examination of the validity of a behavioral mindfulness measure in adults with advanced cancer and their family caregivers

**DOI:** 10.1017/S1478951526103083

**Published:** 2026-07-06

**Authors:** Megan F. Noonan, Catherine E. Mosher, Ekin Secinti, Ashley B. Lewson, Shelley A. Johns

**Affiliations:** 1 Department of Psychology, Indiana University Indianapolis, Indianapolis, IN, USA; 2 Eli Lilly and Company, Indianapolis, IN, USA; 3 Indiana University School of Medicine, Indianapolis, IN, USA; 4 Center for Health Services Research, Regenstrief Institute, Inc., Indianapolis, IN, USA

**Keywords:** Mindfulness, measurement, breath counting task, advanced cancer, family caregivers

## Abstract

**Objectives:**

The breath counting task (BCT) has shown evidence of validity as a behavioral measure of mindfulness in healthy populations but remains largely untested in clinical contexts. The BCT is a computerized measure of present-moment awareness based on breath-counting accuracy. This study provides a preliminary evaluation of its validity in adults with advanced cancer and their family caregivers.

**Methods:**

Fifty-five patient-caregiver dyads were randomized to a 6-week mindfulness intervention or usual care. Participants completed the BCT and self-report surveys at baseline, post-intervention, and 1-month follow-up. The BCT’s construct validity was examined through: (1) sensitivity to mindfulness intervention using linear mixed models, (2) convergent validity via correlations with self-reported mindfulness and theoretically related constructs (e.g., inner peace), and (3) criterion validity via correlations with clinical outcomes (e.g., quality of life).

**Results:**

Findings differed for patients and caregivers. Among caregivers, the BCT demonstrated sensitivity to intervention; breath-counting accuracy on the BCT increased over time in the mindfulness condition and remained stable in the usual care condition. Among patients in the mindfulness condition, greater breath-counting accuracy was moderately associated with better quality of life at follow-ups, including a significant correlation at 1 month (*r* = .57, *p* < .05), supporting its criterion validity. Evidence of convergent validity was limited. However, for patients and caregivers, greater breath-counting accuracy was moderately associated with higher self-reported mindfulness facets following intervention.

**Significance of results:**

Preliminary findings suggest the BCT may capture certain attentional aspects of mindfulness in patients with advanced cancer and caregivers; however, patterns varied across groups, highlighting the need for further evaluation of its validity in clinical contexts.

## Introduction

Mindfulness is awareness of one’s ongoing experiences with an attitude of acceptance and curiosity (Bishop et al. 2004). Monitor and Acceptance Theory (MAT) proposes attention monitoring and acceptance are distinct yet synergistic processes underlying mindfulness-based interventions (MBIs), with their interaction promoting well-being (Lindsay and Creswell 2017). Adults with cancer are the primary medical population in MBI trials (Carlson 2012), with findings of reduced distress and improved coping, quality of life (QOL), and spirituality (Ngamkham et al. 2019; Badaghi et al. 2024).

MBIs are efficacious (Ngamkham et al. 2019; Zhang et al. 2021; Goldberg et al. 2022; Badaghi et al. 2024), but their mechanisms of action are not clear. Mindfulness is regarded as a core mechanism (Carlson 2012), and meta-analyses show moderate increases in self-reported mindfulness following mindfulness training (Visted et al. 2015; Quaglia et al. 2016; Goldberg et al. 2019; Badaghi et al. 2024). However, improvements are inconsistent, with many trials reporting no significant post-intervention gains (Visted et al. 2015; Badaghi et al. 2024). In cancer populations, mindfulness mediated outcomes in only 6 of 11 studies and did not consistently mediate the same outcomes across trials (Carney et al. 2023). Mixed findings may stem from ongoing challenges in defining and measuring mindfulness consistently across studies (Wong et al. 2018; Victorson et al. 2024).

Mindfulness is typically assessed with self-report measures that vary in theoretical foundations and factor structures, limiting comparability across studies (Wong et al. 2018; Baer 2019; Victorson et al. 2024). These measures are prone to response biases, and novices may lack the introspective skill needed to accurately assess their mindfulness (Grossman 2011; Davidson and Kaszniak 2015). These limitations may be exacerbated in cancer populations where cognitive impairment (Wefel et al. 2015), symptom burden (Wu and Harden 2015), and distress (Walbaum et al. 2024) may increase cognitive load and hinder the evaluation of abstract constructs such as mindfulness. Behavioral measures may therefore serve as a valuable complement to self-report by capturing aspects of mindfulness that do not rely on introspective accuracy.

There is growing interest in behavioral measures of mindfulness (Levinson et al. 2014). Several tasks show preliminary evidence of reliability and construct validity. First, the mindful-breathing exercise (Burg and Michalak 2011) involves reporting whether attention is focused on the breath or lost in mind wandering. Second, the mindful awareness task (Hadash and Bernstein 2019) requires verbal labeling of ongoing experience and qualitative coding of responses. Third, the single experience and self-implicit association test (Hadash et al. 2016) uses reaction-time methodology to assess implicit identification with experimentally induced fearful states, which may warrant careful consideration in populations with medical or emotional stress.

Another measure is the breath counting task (BCT), which involves counting one’s breaths while pressing designated computer keys in a fixed sequence (Levinson et al. 2014). Accurate performance is thought to reflect mindfulness because it requires ongoing attention to the present-moment breath and awareness of attentional drift to redirect attention and maintain an accurate count. Compared with other behavioral measures, the BCT has the strongest empirical support (Levinson et al. 2014; Hadash and Bernstein 2019), relies more on objective performance, and involves minimal task demands, making it well suited for medical populations.

The BCT has demonstrated construct validity in nonclinical samples through associations with reduced mind-wandering, greater meta-awareness, and sustained attention (Levinson et al. 2014; Wong et al. 2018). Long-term meditators, who are expected to exhibit greater mindfulness skill, have counted breaths more accurately than age-matched nonmeditators (Levinson et al. 2014), and performance is sensitive to mindfulness training (Isbel et al. 2019, 2020; Djernis et al. 2021). The BCT also shows moderate test–retest reliability over 1 week (ICC = 0.60; Levinson et al. 2014). The measure may align more with attentional than attitudinal components of mindfulness. In healthy samples, it was correlated with attentional measures, the Mindful Attention Awareness Scale and the Acting with Awareness subscale of the Five Facet Mindfulness Questionnaire (FFMQ), but not with attitudinal subscales of the FFMQ, nonjudging and nonreactivity (Ching and Lim 2023).

The BCT’s validity in clinical populations remains largely unexamined. In highly ruminative adolescents, the BCT showed small, nonsignificant associations with anxiety and depressive symptoms (Treves et al. 2025), whereas in nonclinical samples it has shown small to moderate correlations with better psychological well-being (Levinson et al. 2014; Tortella-Feliu et al. 2020), raising questions about its criterion validity across populations.

The BCT may function differently in the context of serious illness where disease-related experiences may differentially impact task performance. Given the widespread use of MBIs in oncology (Carlson 2012; Ngamkham et al. 2019; Xunlin et al. 2020; Badaghi et al. 2024), accurate measurement of mindfulness is essential for determining whether intervention benefits reflect mindfulness-related change rather than nonspecific factors such as social support or facilitator attention. Clarifying the role of mindfulness in MBIs would guide more precise intervention refinement.

This study is a preliminary evaluation of the BCT’s validity in adults with advanced cancer and their family caregivers using secondary data from a randomized trial testing a group-based Mindfulness to Enhance Quality of Life and Support Advance Care Planning (MEANING) intervention (Mosher et al. 2024). MEANING led to significant improvements in patients’ existential well-being and self-efficacy for advance care planning and moderate improvements in caregivers’ QOL and burden compared to usual care. In the current study, the construct validity of the BCT was assessed by (1) examining and comparing intervention effects on BCT scores and self-reported mindfulness, (2) evaluating convergent validity through correlations between the BCT and self-reported mindfulness and theoretically related constructs, and (3) examining criterion validity through the BCT’s associations with clinical outcomes concurrently and over time. Lastly, the same convergent and criterion validity analyses were conducted with the Cognitive and Affective Mindfulness Scale–Revised (CAMS-R) (Feldman et al. 2007). We descriptively explored whether the pattern of associations differed between the BCT, which reflects attentional components of mindfulness, and the CAMS-R, which reflects attentional and attitudinal components. For patients and caregivers, our hypotheses were as follows:

1. The BCT and self-report mindfulness measures are sensitive to change following the MEANING intervention, with a greater impact on the BCT given the limitations of self-report.
2. As evidence of convergent validity, higher breath-counting accuracy on the BCT shows small correlations with self-reported mindfulness and related constructs (i.e., greater acceptance of cancer and inner peace and lower cognitive avoidance).
3. As evidence of criterion validity, higher breath-counting accuracy on the BCT shows small to moderate correlations with clinical outcomes (i.e., lower anxiety and depressive symptoms and better QOL), concurrently and over time.

## Methods

### Study design

This study is a secondary analysis of a randomized controlled pilot trial testing the MEANING intervention which consisted of 6 weekly 2-hour in-person group sessions (Mosher et al. 2024). Patient-caregiver dyads were randomized to MEANING or usual care. Outcomes were assessed at baseline (T1), immediately post-intervention (T2), and 1-month post-intervention (T3). Procedures were approved by the Indiana University Institutional Review Board (IRB#:1702223546).

### Participants

Patients and caregivers were English-speaking adults (≥18 years). Eligible patients had locally advanced or metastatic solid malignancy, life expectancy ≤ 12 months, elevated cognitive avoidance (≥7 on the Mini-Mental Adjustment to Cancer cognitive avoidance subscale; Watson et al. 1994), and a consenting family caregiver. Exclusion criteria included significant functional limitations (>2 on the Eastern Cooperative Oncology Group self-report measure; Bauer et al. 2002), cognitive impairment (≥3 errors on a cognitive screener; Callahan et al. 2002), prior completion of a Physician Orders for Scope of Treatment advance care planning form, or receipt of hospice care.

### Procedure

This trial enrolled 55 patient-caregiver dyads across 4 cohorts between March and September 2017 from Indiana hospitals. Patient eligibility was determined through medical chart review and oncologist consultation. Research staff contacted potentially eligible participants via mailings, telephone calls, and clinic visits. Interested patients identified a caregiver who was then screened for eligibility. At enrollment, eligible dyads provided written informed consent, completed the baseline survey, and were randomized.

The MEANING intervention was led by certified mindfulness teachers and integrated practices from Mindfulness-Based Stress Reduction (Kabat-Zinn 2013, Santorelli and Kabat-Zinn 2013), Interpersonal Mindfulness (Kramer et al. 2008), mindful speaking and listening exercises (Kramer 2007), and education on advance care planning. Usual care participants continued receiving standard oncology care with access to an oncology social worker. After the 1-month follow-up, they attended a session with a mindfulness teacher and received guided audio recordings and information on mindfulness and advance care planning.

### Measures

Participants completed assessments at each timepoint. All self-report measures have evidence of reliability and validity in cancer populations. Measures were identical for patients and caregivers, except for QOL, and the cognitive avoidance and acceptance measures were adapted for caregivers. For all measures, internal consistency was acceptable across time (αs = .70–.94), and higher scores indicate greater levels of the construct.

#### Breath counting task

Behavioral mindfulness was assessed with the BCT, a computerized measure of present-moment awareness based on breath-counting accuracy (Levinson et al. 2014). During a 15-minute session, participants count their breaths from 1 to 9, pressing the down arrow for breaths 1 through 8 and the right arrow for the ninth breath. Staff provided detailed instructions and facilitated a brief practice period. Participants practiced 2 breath count sets of 9 breaths each, and feedback on counting accuracy was delivered at the end of each set to ensure participants understood the task before data collection. BCT accuracy was measured as the percentage of breath cycles correctly counted, with a cycle defined as a full sequence from 1 to 9 entered in the correct order without errors. Higher accuracy reflects greater present-moment awareness.

#### Measures for evaluation of convergent validity

Mindfulness was self-reported using the 5-item Five Facet Mindfulness Questionnaire-Short Form subscales of Acting with Awareness (FFMQ-AA) and Nonreactivity (FFMQ-NR) (Bohlmeijer et al. 2011). Items were rated on a 5-point scale (1 = never or very rarely true; 5 = very often or always true) reflecting experiences over the past month.

Mindfulness was also self-reported using the 10-item Cognitive and Affective Mindfulness Scale-Revised (CAMS-R) (Feldman et al. 2007), which measures attention regulation, orientation to present-moment experience, awareness of experience, and nonjudgmental acceptance. Items were rated on a 4-point scale (1 = rarely/not at all; 4 = almost always).

Acceptance was assessed with the 5-item Peaceful Acceptance of Illness subscale of the Peace, Equanimity, and Acceptance in the Cancer Experience (PEACE) questionnaire (Mack et al. 2008). Items were rated on a 4-point scale (1 = not at all; 4 = to a large extent) and adapted for caregivers.

Inner peace over the past 7 days was assessed with the 4-item Peace subscale of the Functional Assessment of Chronic Illness Therapy-Spiritual Well-Being (FACIT-Sp) scale (Peterman et al. 2002). Items were rated on a 5-point scale (0 = not at all; 4 = very much).

Cognitive avoidance was evaluated with the 4-item Mini-Mental Adjustment to Cancer Cognitive Avoidance subscale (Watson et al. 1994). Items were rated on a 4-point scale (1 = definitely does not apply to me; 4 = definitely applies to me).

**Table 1.** Patient and caregiver characteristics and group comparisons at baseline Table 1 long description.

	Patients			Caregivers		
Characteristics	MEANING (*n* = 33)	Usual care (*n* = 22)	*t*-test/ Chi-square/Fisher’s exact test *p*	MEANING (*n* = 33)	Usual care (*n* = 22)	*t*-test/ Chi-square/Fisher’s exact test *p*
Gender, *n* (%)						
Female	20 (61)	14 (64)	0.82	18 (55)	15 (68)	0.31
Male	13 (39)	8 (36)		15 (45)	7 (32)	
Age						
Mean	64.12	64.09	0.99	60.24	59.38	0.83
SD	9.04	12.55		14.40	13.25	
Range	41.0 − 79.0	37.0 − 82.0		26.0 − 82.0	31.0 − 84.0	
Race and ethnicity, *n* (%)^b^			0.80^a^			1.00^a^
Non-Hispanic White	30 (91)	19 (86)		31 (94)	20 (91)	
Hispanic White	1 (3)	2 (9)		0 (0)	0 (0)	
Non-Hispanic Black	2 (6)	1 (5)		1 (3)	1 (5)	
Hispanic Multiracial	0 (0)	0 (0)		1 (3)	0 (0)	
Employment status, *n* (%)^b^			0.61^a^			0.19^a^
Employed full or part-time	10 (30)	7 (32)		11 (33)	10 (45)	
Retired	15 (46)	11 (50)		16 (49)	7 (32)	
Unemployed due to disability	5 (15)	4 (18)		1 (3)	3 (14)	
Other (e.g., self-employed, homemaker)	3 (9)	0 (0)		5 (15)	1 (5)	
Household income, *n* (%)^b^			0.68^a^			0.38^a^
< $15,000	1 (3)^**b**^	2 (9)		1 (3)	3 (14)	
$15,000–$24,999	5 (15)^**b**^	3 (14)		2 (6)	2 (9)	
$25,000–$49,000	6 (18)^**b**^	7 (32)		11 (33)	3 (14)	
$50,000–$74,999	6 (18)^**b**^	4 (18)		6 (18)	2 (9)	
$75,000–$99,000	5 (15)^**b**^	1 (5)		4 (12)	4 (18)	
>$100,000	9 (27)^**b**^	5 (23)		8 (24)	7 (32)	
Education			0.72^a^			0.62^a^
No bachelor’s degree	14 (42)	11 (50)		15 (46)	11 (50)	
Bachlelor’s degree	6 (18)	5 (23)		5 (15)	3 (14)	
Graduate degree	13 (39)	6 (27)		13 (39)	6 (27)	
Other	0 (0)	0 (0)		0 (0)	1 (5)	
Caregiver relationship to the patient, *n* (%)						0.93^a^
Spouse/partner				22 (67)	13 (59)	
Child				5 (15)	3 (14)	
Sibling				1 (3)	1 (5)	
Parent				0 (0)	1 (5)	
Friend				3 (9)	3 (14)	
Other (e.g., niece, ex-partner)				2 (6)	1 (5)	
Married/living with a partner, *n* (%)	25 (76)	16 (73)	0.80	28 (85)	18 (82)	1.00
Cancer type, *n* (%)			0.39^a^			
Breast	8 (24)	8 (36)				
Prostate	4 (12)	2 (9)				
Colorectal	2 (6)	5 (23)				
Melanoma	3 (9)	2 (9)				
Lung	2 (6)	2 (9)				
Pancreatic	3 (9)	1 (5)				
Other (e.g., ovarian, renal, head/neck, carcinoid)	11 (33)	2 (9)				
Cancer stage, *n* (%)			0.39			
III	6 (18)	1 (5)				
IV	27 (82)	21 (95)				
Treatment status – In treatment, *n* (%)^a^	26 (79)	16 (73)	0.52			
**Outcome measures at baseline, *M* (SD)**						
BCT	0.57 (0.39)	0.70 (0.34)	0.24	0.61 (0.40)	0.79 (0.24)	0.07
FFMQ-Acting with Awareness	3.62 (0.72)	3.97 (0.84)	0.10	3.76 (0.75)	3.95 (0.88)	0.38
FFMQ-NonReactivity	3.39 (0.69)	3.15 (0.96)	0.33	3.13 (0.79)	3.26 (1.00)	0.59
CAMS-R	28.94 (4.93)	30.23 (5.69)	0.38	28.06 (5.66)	27.45 (6.58)	0.72
Acceptance	16.39 (2.76)	17.27 (2.31)	0.22	15.24 (3.13)	15.32 (3.12)	0.93
Peace	10.33 (2.85)	10.91 (3.48)	0.50	9.36 (3.73)	9.41 (3.53)	0.96
Avoidance	8.76 (2.74)	10.52 (2.79)	**0.03**	7.36 (3.21)	8.45 (2.77)	0.20
Anxiety	4.06 (4.30)	2.57 (2.87)	0.17	4.64 (3.77)	3.52 (5.17)	0.37
Depressive symptoms	7.45 (5.12)	4.32 (4.02)	**0.02**	4.91 (4.89)	3.62 (4.20)	0.33
Quality of life	6.54 (1.59)	7.55 (1.28)	**0.02**	2.48 (0.52)	2.60 (0.52)	0.42

MEANING = Mindfulness to Enhance Quality of Life and Support Advanced Care Planning; BCT = Breath counting task; FFMQ = Five Facet Mindfulness Questionnaire; CAMS-R = Cognitive Affective Mindfulness Scale-Revised; Acceptance = PEACE-Acceptance of Illness subscale; Peace = FACIT-Sp-Peace subscale; Avoidance = mini-MAC cognitive avoidance subscale. Significant *p*-values are in bold.

a Monte Carlo simulation was used due to small cell sizes.

b Percents may not add up to 100 due to missing data.

#### Measures for evaluation of criterion validity

Anxiety over the past 2 weeks was assessed using the 7-item Generalized Anxiety Disorder-7 measure (Spitzer et al. 2006). Items were rated on a 4-point scale (0 = not at all; 3 = nearly every day).

Depressive symptoms over the past 2 weeks were assessed using the 8-item Patient Health Questionnaire-8 (Kroenke et al. 2009). Items were rated on a 4-point scale (0 = not at all; 3 = nearly every day).

Patient QOL over the past 2 days was assessed with 13 items from the McGill Quality of Life Questionnaire (Cohen et al. 1997). Subscales measure physical well-being, psychological well-being, existential well-being, and support. Items were rated on a 10-point scale (0 = worst possible situation; 10 = best possible situation) with anchored contrasting statements.

Caregiver QOL over the past 7 days was assessed using the 35-item Caregiver Quality of Life Index-Cancer measure (Weitzner et al. 1999). Items were rated on a 5-point scale (1 = not at all, 5 = very much).

### Data analysis

Data were analyzed using IBM SPSS Statistics (Version 29) and R (R Core Team 2025). Baseline comparisons of study groups were conducted separately for patients and caregivers using *t*-tests, Chi-square tests, and Fisher’s exact tests, as appropriate. Assumptions of normality and homoscedasticity were evaluated. Given the small sample size, emphasis was placed on estimating effect sizes rather than statistical significance.

Construct validity of the BCT was evaluated in 3 ways, with separate analyses for patients and caregivers. First, linear mixed modeling (LMM) was used to examine the impact of the MEANING intervention on the BCT and self-reported mindfulness. Fixed effects included group (MEANING vs. usual care), time (T1, T2, T3; treated as categorical), and their interaction. A significant group  ×  time interaction (*p* < .05) indicated intervention effects. Effect sizes were estimated using partial correlation coefficients with 95% confidence intervals (CIs) (Rosenthal et al. 1994). CIs excluding zero were considered statistically significant for individual measures (Aloe and Thompson 2013). Nonoverlapping CIs for the partial correlation coefficients suggest that the intervention impact differed between the BCT and self-report mindfulness measures.

Second, convergent validity was assessed using cross-sectional correlations between the BCT, self-reported mindfulness, and related constructs at each timepoint. Third, criterion validity was examined using concurrent correlations between the BCT and clinical outcomes (e.g., anxiety) at each timepoint and correlations between baseline BCT scores and clinical outcomes at T2 and T3. All correlations were calculated using Pearson’s *r*, with 2-tailed *p*-values < .05 considered statistically significant.

## Results

### Preliminary analyses

Participant characteristics appear in Table 1. Fifty-five patient-caregiver dyads were randomized to MEANING (*n* = 33) or usual care (*n* = 22). Participant flow is shown in Supplementary Figure 1. Retention did not significantly differ by study condition. Most participants were female, non-Hispanic White, and married or partnered. Patients were typically receiving treatment for stage IV breast or prostate cancer, and most caregivers were spouses. No significant baseline differences were observed between study groups, except MEANING patients reported higher depressive symptoms, and usual care patients reported higher QOL and cognitive avoidance. Descriptive statistics for study variables appear in Supplementary Tables 1 and 2.@@

### Construct validity of the BCT

#### Sensitivity to intervention

LMM results showed no significant group x time interactions for the BCT or self-reported mindfulness for patients or caregivers, except for the BCT in caregivers (Table 2). Means indicated small increases in BCT accuracy over time for MEANING caregivers, whereas scores remained relatively stable for usual care caregivers. Partial correlation coefficients and CIs indicated small, positive intervention effects on the BCT and self-reported mindfulness compared to usual care. Overlapping CIs suggested that intervention impact did not significantly differ between the BCT and self-reported mindfulness.

**Table 2.** Sensitivity of mindfulness measures to intervention: Results from linear mixed model analyses Table 2 long description.

	MEANING intervention						Usual care									
	Baseline		Post- intervention		1 Month post		Baseline	Post-intervention		1 Month post						
Outcome fixed effect	Mean (SE)		Mean (SE)		Mean (SE)		Mean (SE)	Mean (SE)		Mean (SE)		df	*F*	*P*	*Pr*	*95% CI Pr*
Patient outcomes																
Breath counting task	0.57 (0.06)		0.67 (0.07)		0.66 (0.08)		0.70 (0.07)	0.69 (0.08)		0.74 (0.08)						
Group												52	0.76	0.39	0.12	(−0.15, 0.39)
Time												78	0.85	0.43	0.10	(−0.17, 0.38)
Group × time												78	0.62	0.54	0.09	(−0.18, 0.36)
FFMQ-AA	3.62 (0.15)		3.70 (0.16)		3.86 (0.16)		3.97 (0.18)	3.83 (0.19)		4.10 (0.19)						
Group												56	1.29	0.26	0.15	(−0.11, 0.41)
Time												94	2.44	0.09	0.16	(−0.10, 0.42)
Group × time												94	0.57	0.57	0.08	(−0.19, 0.35)
FFMQ-NR	3.39 (0.13)		3.45 (0.15)		3.69 (0.15)		3.16 (0.16)	3.29 (0.17)		3.34(0.17)						
Group												49	2.20	0.14	0.21	(−0.05, 0.46)
Time												91	1.92	0.15	0.14	(−0.12, 0.41)
Group × time												91	0.28	0.76	0.05	(−0.21, 0.32)
CAMS-R	28.94 (0.89)		29.14 (0.97)		29.29 (0.96)		30.23 (1.10)	31.78 (1.13)		32.23 (1.13)						
Group												56	3.27	0.08	0.24	(−0.02, 0.49)
Time												95	1.93	0.15	0.14	(−0.12, 0.40)
Group × time												95	0.99	0.38	0.10	(−0.16, 0.37)
Caregiver outcomes																
Breath counting task	0.62 (0.06)		0.79 (0.07)		0.85 (0.07)		0.79 (0.07)	0.73 (0.08)		0.68 (0.07)						
Group												51	0.06	0.82	0.03	(−0.24, 0.31)
Time												80	0.89	0.42	0.10	(−0.17, 0.38)
Group × time												80	4.95	**0.01**	0.24	(−0.02, 0.50)
FFMQ-AA	3.76 (0.14)		3.74 (0.15)		3.85 (0.15)		3.96(0.17)	3.70 (0.18)		3.72 (0.18)						
Group												56	0.00	0.97	0.01	(−0.26, 0.28)
Time												98	1.14	0.32	0.11	(−0.16, 0.37)
Group × time												98	1.76	0.18	0.13	(−0.13, 0.40)
FFMQ-NR	3.13 (0.15)		3.39 (0.16)		3.21 (0.16)		3.26 (0.19)	3.17 (0.19)		3.13 (0.19)						
Group												55	0.07	0.80	0.03	(−0.23, 0.30)
Time												98	0.60	0.55	0.08	(−0.19, 0.35)
Group × time												98	1.45	0.24	0.12	(−0.14, 0.39)
CAMS-R	28.06 (1.02)		29.27 (1.07)		28.08 (1.07)		27.46 (1.24)	27.41 (1.27)		27.26 (1.27)						
Group												55	0.56	0.46	0.10	(−0.17, 0.37)
Time												96	0.62	0.54	0.08	(−0.19, 0.35)
Group × time												96	0.53	0.59	0.07	(−0.19, 0.34)

The significant *p*-value is in bold. MEANING = Mindfulness to Enhance Quality of Life and Support Advanced Care Planning; FFMQ-AA = Five Facet Mindfulness Questionnaire-Acting with Awareness subscale; FFMQ-NR = Five Facet Mindfulness Questionnaire-Non-Reactivity subscale; CAMS-R = Cognitive Affective Mindfulness Scale-Revised; *Pr =* partial correlation. Patient *n*s = 53–55. Caregiver *n*s = 52–55.

#### Convergent validity

Correlations between the BCT and self-report mindfulness measures were examined. Among MEANING patients, the BCT showed a moderate, positive association with attentional awareness (FFMQ-AA) at follow-ups that was significant at T3 (*r* = .60, *p* < .05, Table 3) and small, nonsignificant associations with other self-report mindfulness measures across timepoints. For usual care patients, most relationships were small and nonsignificant, except for a moderate, nonsignificant correlation with attentional awareness (FFMQ-AA) at T1 (*r* = .40). For MEANING caregivers, the BCT showed moderate, nonsignificant correlations with nonreactivity to internal experiences (FFMQ-NR) at follow-ups; other correlations were small and nonsignificant (Table 3). For usual care caregivers, most associations were also small, except for a moderate, significant association with nonreactivity (FFMQ-NR) at T2 (*r* = .49, *p* < .05).

**Table 3.** Convergent validity: Cross-sectional correlations between the breath counting task, mindfulness measures, and related constructs by study condition Table 3 long description.

	MEANING			Usual care		
	Breath counting task			Breath counting task		
	T1	T2	T3	T1	T2	T3
Patients						
FFMQ-AA	0.22	0.34	0.60*	0.40	−0.00	0.05
FFMQ-NR	0.14	0.09	−0.28	−0.26	−0.02	0.03
CAMS-R	−0.15	0.21	−0.07	−0.14	−0.08	−0.04
Acceptance	−0.33	0.13	−0.10	0.08	0.16	0.21
Peace	−0.20	0.13	−0.02	−0.20	−0.11	0.05
Avoidance	−0.12	− 0.63**	0.06	−0.28	−0.32	−0.27
Caregivers						
FFMQ-AA	0.09	0.06	0.06	−0.01	0.10	− 0.10
FFMQ-NR	0.21	0.36	0.42	0.14	0.49*	0.26
CAMS-R	0.12	0.20	0.29	0.06	0.04	0.01
Acceptance	0.03	0.03	−0.29	0.08	0.44	0.43
Peace	−0.24	0.12	−0.06	0.13	0.32	0.10
Avoidance	−0.30	0.07	−0.09	−0.09	0.35	0.16

Significant correlations are in bold.

* *p* < .05.

** *p* < .01.

FFMQ-AA = Five Facet Mindfulness Questionnaire-Acting with Awareness subscale; FFMQ-NR = Five Facet Mindfulness Questionnaire-Non-Reactivity subscale; CAMS-R = Cognitive Affective Mindfulness Scale-Revised; Acceptance = PEACE-Acceptance of Illness subscale; Peace = FACIT-Sp-Peace subscale; Avoidance = mini-MAC cognitive avoidance subscale; T1 = Baseline; T2 = Post-intervention; T3 = 1 Month post-intervention. MEANING patient *n*s = 16–32. Usual care patient *n*s = 18–20. MEANING caregiver *n*s = 17–31. Usual care caregiver *n*s = 17–18.

Correlations between the BCT and theoretically related constructs were also examined. Among MEANING patients, the BCT showed a significant, negative association with cognitive avoidance at T2 (*r* = − .63, *p* < .01), whereas other correlations were small and nonsignificant (Table 3). Associations were also small and nonsignificant in usual care patients. For caregivers in both study conditions, correlations were generally small and nonsignificant aside from moderate, nonsignificant associations with acceptance at follow-ups among usual care caregivers (*rs* = .44, .43) (Table 3).

#### Criterion validity

Associations between the BCT and clinical outcomes were examined. For MEANING patients, the positive association between the BCT and QOL increased in magnitude over time and was significant at T3 (*r* = .57, *p* < .05; Table 4). Also, for MEANING patients, a moderate, nonsignificant association with depressive symptoms was observed at T2 (*r* = − .44). All other correlations between the BCT and clinical outcomes were small and nonsignificant for patients and caregivers across conditions (Tables 4 and 5).

**Table 4.** Criterion validity: Concurrent and predictive correlations between the breath counting task and clinical outcomes for patients by study condition Table 4 long description.

	MEANING			Usual care		
	BCT T1	BCT T2	BCT T3	BCT T1	BCT T2	BCT T3
Baseline (T1)						
Anxiety	−0.02			−0.28		
Depressive symptoms	0.04			−0.19		
Quality of life	0.04			0.28		
Post-intervention (T2)						
Anxiety	0.17	−0.08		−0.07	0.08	
Depressive symptoms	0.15	−0.44		−0.12	−0.05	
Quality of life	−0.10	0.45		0.12	0.24	
1-month post-intervention (T3)						
Anxiety	−0.19	−0.12	0.03	0.10	−0.03	0.09
Depressive symptoms	−0.11	−0.21	−0.22	0.16	0.31	0.16
Quality of life	−0.22	0.26	0.57*	−0.03	0.05	−0.06

The significant correlation is in bold.

* *p* < .05.

BCT = Breath counting task. MEANING *n*s = 16 − 32. Usual care *n*s = 17 − 20.

**Table 5.** Criterion validity: Concurrent and predictive correlations between the breath counting task and clinical outcomes for caregivers by study condition Table 5 long description.

	MEANING			Usual care		
	BCT T1	BCT T2	BCT T3	BCT T1	BCT T2	BCT T3
Baseline (T1)						
Anxiety	−0.07			−0.02		
Depressive symptoms	0.15			−0.01		
Quality of life	0.04			0.24		
Post-intervention (T2)						
Anxiety	0.30	0.18		0.02	−0.12	
Depressive symptoms	−0.15	−0.03		−0.03	−0.31	
Quality of life	0.06	0.16		0.12	0.39	
1-month post-intervention (T3)						
Anxiety	0.10	−0.11	0.16	−0.09	−0.19	−0.00
Depressive symptoms	−0.03	−0.32	0.04	−0.03	−0.21	−0.10
Quality of life	0.01	0.03	−0.27	0.10	0.23	0.33

BCT = Breath counting task. MEANING *n*s = 17–31. Usual care *n*s = 16–18.

### Comparison of the validity of the BCT and CAMS-R

Compared to the BCT, the CAMS-R showed more consistent and robust correlations with other self-report mindfulness measures and related constructs (Supplementary Tables 3–5). However, associations with cognitive avoidance were generally weak, except for 1 significant correlation for usual care caregivers at T3 (*r* = −.45, *p* < .05). Correlations between the CAMS-R and clinical outcomes were also more consistent than those observed for the BCT across patients and caregivers, with effects tending to be stronger for caregivers.

## Discussion

This study provides the first evaluation of the BCT’s validity in a medical sample, with findings differing between patients with advanced cancer and caregivers. For caregivers, the BCT showed sensitivity to change following a mindfulness intervention, whereas, for MEANING patients, it showed evidence of criterion validity through moderate, positive correlations with QOL. For both patients and caregivers, evidence of the BCT’s convergent validity was limited, although moderate post-intervention associations with mindfulness facets emerged. Consistent with MAT’s emphasis on both attentional and attitudinal components of mindfulness for well-being (Lindsay and Creswell 2017), the BCT–measuring attention monitoring–showed smaller and less consistent associations with clinical outcomes than the CAMS-R, which includes both components. Overall, findings suggest that the BCT has potential as a behavioral measure of present-moment awareness in the context of serious illness.

The BCT’s responsiveness to MBI in caregivers aligns with findings in healthy adults (Isbel et al. 2019, 2020; Djernis et al. 2021), supporting its construct validity. Improvements following mindfulness training suggest the BCT captures changes in the attention monitoring component of mindfulness. The absence of improvement in usual care caregivers further suggests these gains were not due to practice effects. Conversely, self-report mindfulness measures did not significantly change following intervention. Prior evidence has been mixed regarding their sensitivity to change (Visted et al. 2015; Badaghi et al. 2024). Overall, findings raise the possibility that the BCT measures behavioral expressions of mindfulness, particularly attentional components, that may be missed by self-report measures and are less influenced by response bias or limited introspection. Contrary to our hypothesis, the intervention did not have a greater impact on the BCT than on self-reported mindfulness, possibly due to limited statistical power to detect significant differences.

Among patients, neither the BCT nor self-reported mindfulness showed evidence of sensitivity to change following intervention. Patients with advanced cancer may face challenges engaging in mindfulness practices (Lehto and Wyatt 2013; Zimmermann et al. 2018). Cancer-related symptoms such as concentration deficits, fatigue, and physical discomfort (Tate et al. 2018) may interfere with mindfulness practice or breath counting performance. Because the BCT requires sustained attention and coordinated motor responses, these illness-related constraints may limit its ability to detect intervention-related improvements in medically compromised populations.

Prior to mindfulness training, correlations between the BCT and self-reported mindfulness were generally small, with moderate associations emerging only after intervention, consistent with limited prior findings (Isbel et al. 2020). For patients, the BCT aligned with attentional awareness (FFMQ-AA), whereas for caregivers it aligned with nonreactivity to internal experiences (FFMQ-NR). These patterns may reflect group-specific burdens that impact attention monitoring. Patients’ symptoms may disrupt deliberate attentional awareness in daily activities, increasing reliance on “autopilot” functioning, as reflected in the FFMQ-AA. Caregivers’ emotional demands may impair sustained attention, whereas the opposite tendency (e.g., perceiving thoughts and feelings without getting lost in them) is captured by the FFMQ-NR. Mindfulness training may have supported both groups in reallocating attention to the present moment and improved the accuracy of their self-reported mindfulness, increasing convergence between behavioral and subjective indicators. The isolated significant association between the BCT and nonreactivity to internal experiences (FFMQ-NR) in usual care caregivers did not increase in magnitude over time, suggesting that convergence may depend on mindfulness training.

The BCT was not significantly associated with CAMS-R scores among patients or caregivers in either condition. The CAMS-R assesses multiple mindfulness facets, which may dilute associations with more specific attentional processes measured by the BCT. The CAMS-R has not been previously examined with the BCT, but prior work shows that attentional mindfulness measures better correlate with the BCT (Ching and Lim 2023).

Findings provide limited evidence of convergence between the BCT and theoretically related psychological processes. For MEANING patients, the BCT was associated with decreased cognitive avoidance only at follow-up. For caregivers, this association was weak across timepoints. Associations with acceptance of cancer and inner peace were minimal and inconsistent across timepoints and groups, suggesting limited overlap between these attitudinal and spiritually oriented constructs and breath counting performance. The CAMS-R showed more consistent associations with these constructs, indicating better conceptual alignment.

The BCT showed some evidence of criterion validity through convergence with clinical outcomes. Among patients, the magnitude of its association with QOL increased over time following intervention, consistent with prior findings linking BCT performance to better psychological well-being in healthy samples (Levinson et al. 2014; Tortella-Feliu et al. 2020). These associations did not emerge for caregivers, possibly due to differences in QOL measurement: the patient measure assesses broad QOL domains, whereas the caregiver measure includes caregiving-specific burden items that may be less related to attention regulation. Consistent with findings in clinical samples (Treves et al. 2025), the BCT was generally unrelated to depressive symptoms or anxiety in patients and caregivers. In contrast, the CAMS-R showed more robust associations with clinical outcomes for patients and caregivers, aligning with MAT (Lindsay and Creswell 2017), which posits attention monitoring and acceptance are necessary for improvements in well-being.

Overall, findings suggest that the BCT has potential as a measure of attentional features of mindfulness in the context of serious illness, with mixed evidence across groups. The BCT demonstrated sensitivity to change among caregivers, associations with QOL in patients, and correlations with different self-reported mindfulness facets across groups.

### Limitations and future directions

This study has several limitations. The relatively small sample size reduced statistical power to detect significant effects. The sample was primarily non-Hispanic White and recruited from oncology clinics in Indiana. Data were drawn from a randomized controlled trial that required weekly in-person sessions; thus, results may not generalize to patients or caregivers with greater illness burden or reduced ability to participate in research.

Future research should prioritize larger, adequately powered studies to more precisely evaluate the BCT in medical contexts. Studies with more demographically and clinically diverse samples are needed to enhance generalizability. Additionally, longitudinal studies may examine whether baseline BCT performance predicts subsequent psychological outcomes and the degree to which illness-related burdens moderate these relationships.

Future research may also evaluate convergence of the BCT with behavioral measures of attentional processes such as sustained attention, mind-wandering, and meta-awareness (Levinson et al. 2014; Wong et al. 2018). Prior work in healthy samples suggests the BCT is distinct from broader cognitive constructs such as working memory capacity and sustained attention (Levinson et al. 2014); testing whether this distinction holds in medical populations would clarify whether the BCT reflects mindfulness-related processes rather than general cognitive performance.

The BCT remains the most widely used behavioral measure of present-moment awareness and has accumulating evidence supporting its validity (Levinson et al. 2014; Wong et al. 2018; Isbel et al. 2020; Ching and Lim 2023). Several alternative behavioral measures of mindfulness show preliminary promise (Burg and Michalak 2011; Hadash et al. 2016; Hadash and Bernstein 2019), but remain largely untested in medical populations and have not been evaluated for sensitivity to mindfulness intervention. The relative simplicity of the BCT may make it particularly suitable for patients with cancer and caregivers who often face cognitive and emotional strain. Continued validation in adequately powered and diverse samples will help clarify its validity. Incorporating attentional behavioral measures may strengthen evidence for construct validity but should be implemented judiciously to minimize participant burden. Using the BCT alongside self-reported mindfulness may provide a more comprehensive understanding of the mechanisms underlying MBIs and inform refinements to optimize their efficacy.

## Supporting information

10.1017/S1478951526103083.sm001 Noonan et al. supplementary material Noonan et al. supplementary material

## Supplementary material

The supplementary material for this article can be found at https://doi.org/10.1017/S1478951526103083.

## Table 1 Long description

Baseline characteristics and outcome measures are compared between MEANING and usual care for patients with advanced cancer and their caregivers. Patient demographics were similar across groups: about three in five were female, average age was about 64 years, most were non-Hispanic White, and most were married or living with a partner; cancer stage was predominantly stage four in both groups. Caregiver demographics were also similar, with average age about 60 years and most caregivers being spouses or partners. For patient mindfulness-related measures (breath counting, mindfulness questionnaires, acceptance, and peace), group averages were close and statistical tests did not indicate clear differences. Patient avoidance was lower in MEANING than usual care, while depressive symptoms were higher and quality of life was lower in MEANING than usual care; these were the main baseline differences flagged by the statistical tests. Caregiver outcome measures (including breath counting, mindfulness scales, acceptance, peace, avoidance, anxiety, depressive symptoms, and quality of life) were broadly comparable between groups, with no clear differences indicated. Some tests used simulation methods because of small category counts, and percentages may not total 100 due to missing data.

Navigate back to Table 1.

## Table 2 Long description

The table reports patient and caregiver mindfulness outcomes for MEANING versus usual care at baseline, immediately after the program, and one month later, with model tests for group, time, and their combined effect. Outcomes include a breath-counting task plus three questionnaire measures: acting with awareness, non-reactivity, and a general mindfulness scale. For patients, mean scores changed modestly over time in both groups, and none of the group, time, or combined effects reached statistical significance across outcomes. For caregivers, breath-counting increased in the MEANING group from about 0.62 at baseline to about 0.85 at one month, while usual care decreased from about 0.79 to about 0.68; the combined group-and-time effect was statistically significant with a small-to-moderate partial correlation and a confidence interval that nearly touches zero. Caregiver questionnaire scores showed small fluctuations without statistically significant effects for group, time, or their combination. Overall, the main detectable intervention-related change is limited to caregiver breath-counting, and other measures do not show clear differential improvement between groups. Interpret results cautiously because sample sizes vary slightly by measure and time point and most effects are small with confidence intervals spanning no effect.

Navigate back to Table 2.

## Table 3 Long description

The table reports cross-sectional correlations between breath counting task scores and mindfulness-related measures for patients and caregivers in two study conditions, measured at baseline, post-intervention, and one month later. For MEANING patients, the strongest positive association is with acting with awareness at one month (correlation 0.60, statistically significant), while the strongest negative association is with cognitive avoidance at post-intervention (correlation minus 0.63, statistically significant). Other MEANING patient correlations are small to moderate and vary in direction, including generally weak links with non-reactivity, CAMS-R, acceptance, and peace across time points. In usual care patients, correlations are mostly small and none are marked as statistically significant; acting with awareness is moderate at baseline (0.40) but near zero at later time points. For caregivers in MEANING, correlations are generally small to moderate and none are marked as statistically significant, with non-reactivity reaching 0.42 at one month. For caregivers in usual care, the only statistically significant result is a positive association between breath counting and non-reactivity at post-intervention (0.49). Overall, statistically significant relationships are few and differ by group and time point, and sample sizes vary across cells, so patterns should be interpreted cautiously.

Navigate back to Table 3.

## Table 4 Long description

The table reports correlations between Breath Counting Task scores at three time points and clinical outcomes (anxiety, depressive symptoms, quality of life) measured at baseline, post-intervention, and one month later, shown separately for MEANING and Usual Care. In the MEANING group, the only statistically significant association is between breath counting at one month and quality of life at one month, a moderate positive correlation of 0.57. Other MEANING correlations are small to moderate and mixed in direction, such as post-intervention breath counting with depressive symptoms at post-intervention (negative 0.44) and with quality of life at post-intervention (positive 0.45), but these are not marked significant. In Usual Care, correlations are generally small across outcomes and time points, with the largest around 0.31 between breath counting at post-intervention and depressive symptoms at one month, and none are indicated as significant. Baseline outcome correlations with baseline breath counting are near zero in MEANING and modestly negative for anxiety and depressive symptoms in Usual Care. Blank cells indicate correlations not provided for that pairing. Correlations describe association only and do not establish causation, and sample sizes differ by condition.

Navigate back to Table 4.

## Table 5 Long description

The table reports correlations between breath counting task scores at three time points and caregiver outcomes (anxiety, depressive symptoms, and quality of life) measured at baseline, post-intervention, and one month later, shown separately for the MEANING and usual care groups. At baseline, correlations are near zero in both groups, with the largest being a small positive link between breath counting and quality of life in usual care (about 0.24). At post-intervention, MEANING shows a moderate positive association between baseline breath counting and anxiety (about 0.30), while usual care shows the clearest positive association between breath counting and quality of life, strongest for the second breath counting time point (about 0.39). At one month, MEANING shows its strongest negative association between breath counting at the second time point and depressive symptoms (about minus 0.32), and a negative association between breath counting at the third time point and quality of life (about minus 0.27). In usual care at one month, quality of life remains positively related to breath counting, reaching about 0.33 for the third breath counting time point, while anxiety and depressive symptoms correlations are small and mostly negative. Overall, most relationships are small, directions vary by outcome and timing, and the pattern differs between study conditions. Interpretation should be cautious because sample sizes vary by condition and are relatively small.

Navigate back to Table 5.
