# Supplementary material for: Preliminary examination of the validity of a behavioral mindfulness measure in adults with advanced cancer and their family caregivers
Source: Palliat Support Care. 2026 Jul 6;24:e187. doi: 10.1017/S1478951526103083 (PMC13370295; doi:10.1017/S1478951526103083)
Supplement: Noonan et al. supplementary material [file S1478951526103083sup001.docx]

**Preliminary Examination of the Validity of a Behavioral Mindfulness Measure in Adults with Advanced Cancer and Their Family Caregivers: Supplementary Tables**

**Supplementary Figure 1. Participant flow diagram**

Consented

(*n=*55 patients*; n=*55 caregivers)

Approached (*n=*315 patients)

Screened (*n=*214 patients)

Baseline assessment

(*n=*55 patients*; n=*55 caregivers)

Randomized

(*n=*55 patients*; n=*55 caregivers)

***Usual Care***

(*n=*22 patients*; n=*22 caregivers)

Post-intervention assessment

(*n=*20 patients*; n=*20 caregivers)

1-month post-intervention assessment

(*n=*22 patients*; n=*22 caregivers)

Excluded (*n*=159 patients)

- Eligible, not enrolled (*n*=60)
- Ineligible (*n*=99)

Refused screening (*n*=101 patients)

***MEANING***

(*n=*33 patients*; n=*33 caregivers)

Post-intervention assessment

(*n=*26 patients*; n=*28 caregivers)

1-month post-intervention assessment

(*n=*26 patients*; n=*27 caregivers)

MEANING = Mindfulness to Enhance Quality of Life and Support Advance Care Planning

| Supplementary Table 1. Descriptive statistics for patient variables by study condition | | | | | | |
| --- | --- | --- | --- | --- | --- | --- |
|  | MEANING | | | Usual Care | | |
|  | Means (SD) | | | Means (SD) | | |
|  | T1 | T2 | T3 | T1 | T2 | T3 |
| BCT | 0.57 (0.39) | 0.62 (0.32) | 0.66 (0.32) | 0.70 (0.34) | 0.68 (0.30) | 0.73 **(**0.32**)** |
| FFMQ-AA | 3.62 (0.72) | 3.65 (1.01) | 3.81 (1.04) | 3.97 (0.84) | 3.76 (0.97) | 4.03 (0.81) |
| FFMQ-NR | 3.39 (0.69) | 3.49 (0.75) | 3.71 (0.73) | 3.15 (0.96) | 3.34 (0.75) | 3.39 (0.83) |
| CAMS-R | 28.94 (4.93) | 29.24 (4.99) | 29.38 (4.96) | 30.23 (5.69) | 31.60 (5.66) | 32.05 (5.81) |
| Acceptance | 16.39 (2.76) | 17.40 (2.36) | 17.54 (2.69) | 17.27 (2.31) | 17.30 (2.25) | 17.65 (2.23) |
| Peace | 10.33 (2.85) | 11.56 (2.90) | 11.77 (3.48) | 10.91 (3.48) | 12.15 (2.72) | 12.00 (2.81) |
| Avoidance | 8.76 (2.74) | 8.64 (2.40) | 8.46 (3.17) | 10.52 (2.79) | 10.10 (3.35) | 9.95 (2.87) |
| Anxiety | 4.06 (4.30) | 3.00 (3.45) | 3.77 (4.80) | 2.57 (2.87) | 2.53 (3.52) | 2.37 (2.69) |
| Depressive symptoms | 7.45 (5.12) | 5.52 (4.97) | 6.88 (5.90) | 4.32 (4.02) | 4.10 (4.14) | 3.26 (3.31) |
| Quality of life | 6.54 (1.59) | 7.34 (1.37) | 7.30 (1.54) | 7.55 (1.28) | 7.50 (1.36) | 7.92 (1.22) |
|  |  |  |  |  |  |  |
| MEANING = Mindfulness to Enhance Quality of Life and Support Advanced Care Planning; BCT = Breath Counting Task; FFMQ-AA = Five Facet Mindfulness Questionnaire-Acting with Awareness subscale; FFMQ-NR = Five Facet Mindfulness Questionnaire-Non-Reactivity subscale; CAMS-R = Cognitive Affective Mindfulness Scale-Revised; Acceptance = PEACE-Acceptance of Illness subscale; Peace = FACIT-Sp-Peace subscale; Avoidance = mini-MAC cognitive avoidance subscale; T1 = Baseline; T2 = Post-Intervention; T3 = 1 Month Post-Intervention. MEANING *ns* = 16-33. Usual care *ns* = 18-22. | | | | | | |

| Supplementary Table 2. Descriptive statistics for caregiver variables by study condition | | | | | | |
| --- | --- | --- | --- | --- | --- | --- |
|  | MEANING | | | Usual Care | | |
|  | Means (SD) | | | Means (SD) | | |
|  | T1 | T2 | T3 | T1 | T2 | T3 |
| BCT | 0.61 (0.40) | 0.76 (0.29) | 0.83 (0.25) | 0.79 (0.24) | 0.73 (0.35) | 0.68 (0.34) |
| FFMQ-AA | 3.76 (0.75) | 3.79 (0.69) | 3.90 (0.78) | 3.95 (0.88) | 3.66 (0.80) | 3.68 (1.17) |
| FFMQ-NR | 3.13 (0.79) | 3.41 (0.84) | 3.24 (0.93) | 3.26 (1.00) | 3.13 (0.86) | 3.09 (1.06) |
| CAMS-R | 28.06 (5.66) | 29.78 (4.99) | 28.59 (5.81) | 27.45 (6.58) | 27.55 (6.33) | 27.4 (6.64) |
| Acceptance | 15.24 (3.13) | 16.04 (2.59) | 16.15 (2.84) | 15.32 (3.12) | 15.35 (3.57) | 15.75 (2.90) |
| Peace | 9.36 (3.73) | 10.57 (3.18) | 10.41 (3.79) | 9.41 (3.53) | 9.20 (3.81) | 9.16 (3.88) |
| Avoidance | 7.36 (3.21) | 7.15 (2.58) | 7.44 (2.33) | 8.45 (2.77) | 8.90 (2.75) | 9.30 (2.92) |
| Anxiety | 4.64 (3.77) | 3.76 (3.35) | 3.85 (3.77) | 3.52 (5.17) | 4.95 (5.56) | 4.40 (6.19) |
| Depressive symptoms | 4.91 (4.90) | 3.58 (4.07) | 3.78 (3.51) | 3.62 (4.20) | 5.90 (5.44) | 4.65 (5.59) |
| Quality of life | 2.48 (0.52) | 2.51 (0.43) | 2.76 (0.42) | 2.60 (0.52) | 2.62 (0.42) | 2.69 (0.51) |
|  |  |  |  |  |  |  |
| MEANING = Mindfulness to Enhance Quality of Life and Support Advanced Care Planning; BCT = Breath Counting Task; FFMQ-AA = Five Facet Mindfulness Questionnaire-Acting with Awareness subscale; FFMQ-NR = Five Facet Mindfulness Questionnaire-Non-Reactivity subscale; CAMS-R = Cognitive Affective Mindfulness Scale-Revised; Acceptance = PEACE-Acceptance of Illness subscale; Peace = FACIT-Sp-Peace subscale; Avoidance = mini-MAC cognitive avoidance subscale; T1 = Baseline; T2 = Post-Intervention; T3 = 1 Month Post-Intervention. MEANING *ns* = 17-33. Usual care *ns* = 17-22. | | | | | | |

| Supplementary Table 3. Convergent Validity: Cross-sectional correlations between the CAMS-R, mindfulness measures, and related constructs for patients by study condition | | | | | | |
| --- | --- | --- | --- | --- | --- | --- |
|  | MEANING | | | Usual Care | | |
|  | CAMS-R | | | CAMS-R | | |
|  | T1 | T2 | T3 | T1 | T2 | T3 |
| **Patients** |  |  |  |  |  |  |
| FFMQ-AA | **0.50^**^** | **0.42^*^** | **0.49^*^** | 0.37 | **0.45^*^** | **0.69**^**^ |
| FFMQ-NR | 0.34 | 0.19 | 0.38 | 0.27 | **0.64^**^** | 0.19 |
| Acceptance | **0.53^**^** | 0.03 | 0.38 | **0.50^*^** | **0.48^*^** | **0.58^**^** |
| Peace | **0.58**^**^ | **0.75^**^** | **0.51^**^** | **0.62^**^** | **0.69^**^** | **0.78^**^** |
| Avoidance | -0.05 | -0.19 | -0.18 | -0.11 | -0.18 | -0.21 |
|  |  |  |  |  |  |  |
| **Caregivers** |  |  |  |  |  |  |
| FFMQ-AA | **0.52^**^** | **0.66^**^** | **0.74^**^** | **0.71^**^** | **0.75^**^** | **0.84^**^** |
| FFMQ-NR | **0.39^*^** | **0.68^**^** | **0.52^**^** | **0.48^*^** | 0.32 | 0.39 |
| Acceptance | **0.55^**^** | **0.54^**^** | **0.54^**^** | **0.69^**^** | **0.62^**^** | **0.74^**^** |
| Peace | **0.56^**^** | **0.72^**^** | **0.67^**^** | 0.40 | **0.81^**^** | **0.80^**^** |
| Avoidance | -0.07 | -0.22 | -0.27 | -0.29 | -0.29 | **-0.45^*^** |
|  |  |  |  |  |  |  |
| Significant correlations are in bold. **p* < .05. ***p* < .01. CAMS-R = Cognitive Affective Mindfulness Scale-Revised; MEANING = Mindfulness to Enhance Quality of Life and Support Advanced Care Planning; FFMQ-AA = Five Facet Mindfulness Questionnaire-Acting with Awareness subscale; FFMQ-NR = Five Facet Mindfulness Questionnaire-Non-Reactivity subscale; Acceptance = PEACE-Acceptance of Illness subscale; Peace =FACIT-Sp-Peace subscale; Avoidance = mini-MAC cognitive avoidance subscale; T1 = Baseline; T2 = Post-Intervention; T3 = 1 Month Post-Intervention. MEANING patient *n*s = 24-33. Usual care patient *n*s = 20-22. MEANING caregiver *n*s = 27-33. Usual care caregiver *n*s = 19-22. | | | | | | |

| Supplementary Table 4. Criterion Validity: Cross-sectional and predictive correlations between the CAMS-R and clinical outcomes for patients by study condition | | | | | | |
| --- | --- | --- | --- | --- | --- | --- |
|  | MEANING | | | Usual Care | | |
|  | CAMS-R T1 | CAMS-R T2 | CAMS-R T3 | CAMS-R T1 | CAMS-R T2 | CAMS-R T3 |
| **Baseline (T1)** |  |  |  |  |  |  |
| Anxiety | -0.32 |  |  | -0.35 |  |  |
| Depressive symptoms | -0.26 |  |  | -**0.44^*^** |  |  |
| Quality of life | 0.19 |  |  | 0.32 |  |  |
|  |  |  |  |  |  |  |
| **Post-intervention (T2)** |  |  |  |  |  |  |
| Anxiety | **-0.52^**^** | **-0.49^*^** |  | -0.40 | **-0.58^**^** |  |
| Depressive symptoms | 0.01 | 0.02 |  | -0.07 | -0.36 |  |
| Quality of life | 0.35 | 0.34 |  | 0.27 | **0.57^**^** |  |
|  |  |  |  |  |  |  |
| **1-month post-intervention (T3)** |  |  |  |  |  |  |
| Anxiety | 0.02 | -0.04 | -0.10 | **-0.53^*^** | **-0.55^*^** | **-0.67^**^** |
| Depressive symptoms | 0.06 | 0.13 | 0.03 | -0.09 | -0.27 | -0.44 |
| Quality of life | 0.24 | 0.37 | 0.32 | 0.18 | 0.39 | **0.48^*^** |
|  |  |  |  |  |  |  |
| Significant correlations are in bold. **p* < .05. ***p* < .01. CAMS-R = Cognitive Affective Mindfulness Scale-Revised; MEANING = Mindfulness to Enhance Quality of Life and Support Advanced Care Planning; T1 = Baseline; T2 = Post-Intervention; T3 = 1 Month Post-Intervention. MEANING *n*s = 24-33. Usual care *n*s = 19-22. | | | | | | |

| Supplementary Table 5. Criterion Validity: Cross-sectional and predictive correlations between the CAMS-R and clinical outcomes for caregivers by study condition | | | | | | |
| --- | --- | --- | --- | --- | --- | --- |
|  | MEANING | | | Usual Care | | |
|  | CAMS-R T1 | CAMS-R T2 | CAMS-R T3 | CAMS-R T1 | CAMS-R T2 | CAMS-R T3 |
| **Baseline (T1)** |  |  |  |  |  |  |
| Anxiety | **-0.62^**^** |  |  | **-0.62^**^** |  |  |
| Depressive symptoms | **-0.56^**^** |  |  | **-0.54^*^** |  |  |
| Quality of life | **0.42^*^** |  |  | 0.32 |  |  |
|  |  |  |  |  |  |  |
| **Post-intervention (T2)** |  |  |  |  |  |  |
| Anxiety | -0.38 | **-0.56^**^** |  | **-0.61^**^** | **-0.61^**^** |  |
| Depressive symptoms | -0.39 | -0.36 |  | **-0.45^*^** | -0.44 |  |
| Quality of life | **0.49^**^** | **0.49^**^** |  | 0.31 | **0.54^*^** |  |
|  |  |  |  |  |  |  |
| **1-month post-intervention (T3)** |  |  |  |  |  |  |
| Anxiety | -0.15 | **-0.47^*^** | **-0.58^**^** | **-0.55^*^** | **-0.49^*^** | **-0.72^**^** |
| Depressive symptoms | **-0.38^*^** | **-0.51^**^** | **-0.64^**^** | **-0.47^*^** | -0.34 | **-0.66^**^** |
| Quality of life | **0.46^*^** | **0.60^**^** | **0.59^**^** | 0.39 | 0.36 | **0.66^**^** |
|  |  |  |  |  |  |  |
| Significant correlations are in bold. **p* < .05. ***p* < .01. CAMS-R = Cognitive Affective Mindfulness Scale-Revised; MEANING = Mindfulness to Enhance Quality of Life and Support Advanced Care Planning; T1 = Baseline; T2 = Post-Intervention; T3 = 1 Month Post-Intervention. MEANING *n*s = 25-33. Usual care *n*s = 20-21. | | | | | | |
